# Transcriptomic and Metabolomic Analysis Unravels the Molecular Regulatory Mechanism of Fatty Acid Biosynthesis in *Styrax tonkinensis* Seeds under Methyl Jasmonate Treatment

**DOI:** 10.3390/ijms23116190

**Published:** 2022-05-31

**Authors:** Chen Chen, Hong Chen, Chao Han, Zemao Liu, Ming Ni, Qikui Wu, Fangyuan Yu

**Affiliations:** 1 Collaborative Innovation Centre of Sustainable Forestry in Southern China, College of Forest Science, Nanjing Forestry University, 159 Longpan Road, Nanjing 210037, China; cc0212@njfu.edu.cn (C.C.); hongchen@njfu.edu.cn (H.C.); hanc0909@njfu.edu.cn (C.H.); 727046890@njfu.edu.cn (Z.L.); niming@njfu.edu.cn (M.N.); 2 State Forestry and Grassland Administration Key Laboratory of Silviculture in Downstream Areas of the Yellow River, College of Forestry, Shandong Agricultural University, Tai’an 271018, China; qkwu@sdau.edu.cn

**Keywords:** fatty acid biosynthesis, metabolome, methyl jasmonate, *Styrax tonkinensis*, transcriptome

## Abstract

As the germ of a highly productive oil tree species, *Styrax tonkinensis* seeds have great potential to produce biodiesel and they have marvelous fatty acid (FA) composition. In order to explore the molecular regulatory mechanism of FA biosynthesis in *S. tonkinensis* seeds after methyl jasmonate (MJ) application, transcriptomic and metabolomic techniques were adopted so as to dissect the genes that are related to FA biosynthesis and their expression levels, as well as to discover the major FA concentration and composition. The results revealed that 200 μmol/L of MJ (MJ200) increased the crude fat (CF) mass fraction and generated the greatest impact on CF accumulation at 70 days after flowering. Twenty FAs were identified, among which palmitic acid, oleic acid, linoleic acid and linolenic acid were the major FAs, and the presence of MJ200 affected their concentrations variously. MJ200 could enhance FA accumulation through elevating the activity of enzymes that are related to FA synthesis. The number of differentially expressed genes increased with the seeds’ development in general. Fatty acid biosynthesis, the biosynthesis of unsaturated fatty acid, fatty acid elongation and glycerolipid metabolism were the main lipid metabolism pathways that were found to be involved. The changes in the expression levels of *EAR*, *KAR*, *accA*, *accB* and *SAD2* were consistent with the changes in the CF mass fraction, indicating that they are important genes in the FA biosynthesis of *S. tonkinensis* seeds and that MJ200 promoted their expression levels. In addition, *bZIP* (which was screened by weighted correlation network analysis) also created significant impacts on FA biosynthesis. Our research has provided a basis for further studies on FA biosynthesis that is regulated by MJ200 at the molecular level and has helped to clarify the functions of key genes in the FA metabolic pathway in *S. tonkinensis* seeds.

## 1. Introduction

Lipids are a large family of biomolecules including triglycerides, which are formed from three molecules of fatty acids (FAs) and one molecule of glycerol [1]. Plant seeds, especially oil plant seeds, are rich in lipids. Lipid synthesis is a complex physiological and biochemical process, one which is closely related to the metabolism of storages in seeds and affected by environmental factors [2,3,4]. In recent years, with the development of the biodiesel industry, the research into using seed oil as the raw material for biodiesel production has attracted great attention from scientists all over the world. FA is the main component of lipids and its synthesis is mainly carried out in the plastid. FA de novo synthesis begins with the conversion of acetyl-CoA to malonyl-CoA (Mal-CoA), which is catalyzed by acetyl-CoA carboxylase (ACC). Then, Mal-CoA:ACP S-malonyltransferase (MAT), the principal substrate for the subsequent elongation, transfers Mal-CoA to the malonyl group. Next, the carbon chain is extended by the FA synthases system through condensation, reduction and dehydration [5]. FAs have practical values in chemical and pharmaceutical industries and could be utilized as crucial raw materials for food processing [6,7]. Many unsaturated FAs (UFAs) are in high demand and cannot be synthesized by humans. They can regulate blood lipids, enhance immunity, prevent cardiovascular diseases and so on [8]. To date, researchers have conducted extensive studies on the lipid content, FA compositions and molecular mechanisms of FA biosynthesis in plant seeds. Studies on the biosynthesis and accumulation of FAs that are associated with different enzyme genes are of great significance for understanding the interactions between multiple genes and further illuminating their genetic regulatory networks [9]. ACC catalyzes the carboxylation of acetyl CoA to form malonyl CoA, which is a pivotal regulatory step for FA biosynthesis and lipid formation, while FA desaturases (FADs) are the important enzymes that create double bonds in FA acyl chains in a stepwise manner, starting from stearic acid [10,11]. Taylor et al. [12] have demonstrated that the expression of diacylglycerol acyltransferase (*DGAT*) affected seed oil content and FA composition. The overexpression of *AtDGAT1* made the DGAT activity in transgenic *Arabidopsis thaliana* seeds 10% to 70% higher than that of the wild type and the oil content was also higher in the transgenic plant [13]. DNA binding with one finger (*Dof*) is highly conserved and can regulate the transcription of FA biosynthesis-related genes in lipid metabolism. The heterologous expression of *GmDof4* or *GmDof11* has been found to have increased the FA content in *A. thaliana* seeds by 11–24% [14]. In the basic leucine zipper (*bZIP*) gene family, the heterologous expression of *GmbZIP123* contributed to FA accumulation in *A. thaliana* seeds [15]. The overexpression of Wrinkled1 (*WRI1*) in *A. thaliana* and *Zea mays* resulted in a significant increase in the oil content of these transgenic plants [16,17,18]. *Jatropha curcas* and *Vernicia fordii* are important economic tree species with oil contents that are close to 40% [19,20]. *Moringa oleifera* seeds possessed ten FAs, among which oleic acid was the most abundant FA, followed by palmitic acid [21].

*Styrax tonkinensis* is a fast-growing tree species with strong adaptability and it is widely distributed in Laos, Vietnam, Thailand and southern China [22,23]. It blooms in mid-May and has great ornamental value [24]. The flowers of *S. tonkinensis* are present in racemes with a light fragrance and can be used as medicine to relieve pain [25]. The benzoinum that is extracted from the dry resin of *S. tonkinensis* could promote anti-antioxidant, anti-inflammatory and anti-complement activity [26]. Previous reports have showed that *S. tonkinensis* seeds contain abundant oil and the UFA content accounted for more than 80% of the total FA content when the seeds matured [27]. The molecular mechanism of FA biosynthesis in *S. tonkinensis* seeds was elucidated by using transcriptomic analysis [28]. Although *S. tonkinensis* is a biodiesel species with high oil content, the crude fat (CF) mass fraction in the seeds has a rapid declination period and the FA compositions need to be further optimized in order to realize the industrialization of *S. tonkinensis*.

Commonly, adopting cultivation techniques, such as spraying plant exogenous growth regulators, is a feasible way to improve oil accumulation in plant seeds. The application of 24-epibrassinolide on different varieties of *Carthamus tinctorius* significantly increased the content of FA in the seeds [29]. Salicylic acid and jasmonic acid elevated *Glycine max* seeds’ FA content and the seeds’ FA composition also changed drastically. Mainly, the content of oleic acid was reduced, while the content of linoleic acid and linolenic acid was enhanced [30]. As a derivative of jasmonic acid, methyl jasmonate (MJ) was first isolated from *Jasminum grandiflorum* and its physiological effects on plants are similar to those of jasmonic acid [31]. MJ is a signal molecule that is widely present in higher plants and has been reported to play an important role in the regulation of signal transduction in plant defense genes [32]. MJ has been extensively utilized for enhancing plants’ photosynthesis, improving their floral composition, ameliorating their fruit quality and so on [33,34,35]. In addition, MJ was verified to contribute to FA accumulation [36,37].

So far, the effects of exogenous plant growth regulators on the seed oil accumulation of *S. tonkinensis* have not been reported. Therefore, we applied exogenous MJ on *S. tonkinensis* and collected seeds on different days after flowering (DAF). Physiological determination, transcriptome and metabolome analysis were combined in order to: (1) probe the impact of MJ on the dynamic of the CF mass fraction and FA composition in *S. tonkinensis* seeds; (2) identify the genes that are related to FA biosynthesis and their expression patterns and (3) reveal the possible physiological and molecular regulatory mechanism of FA biosynthesis under exogenous MJ treatment.

## 2. Results

### 2.1. Dynamic Changing Trend of Crude Fat Mass Fraction and Fatty Acid Synthesis-Related Enzymes Activity under MJ200 Treatment

**Figure 1:**
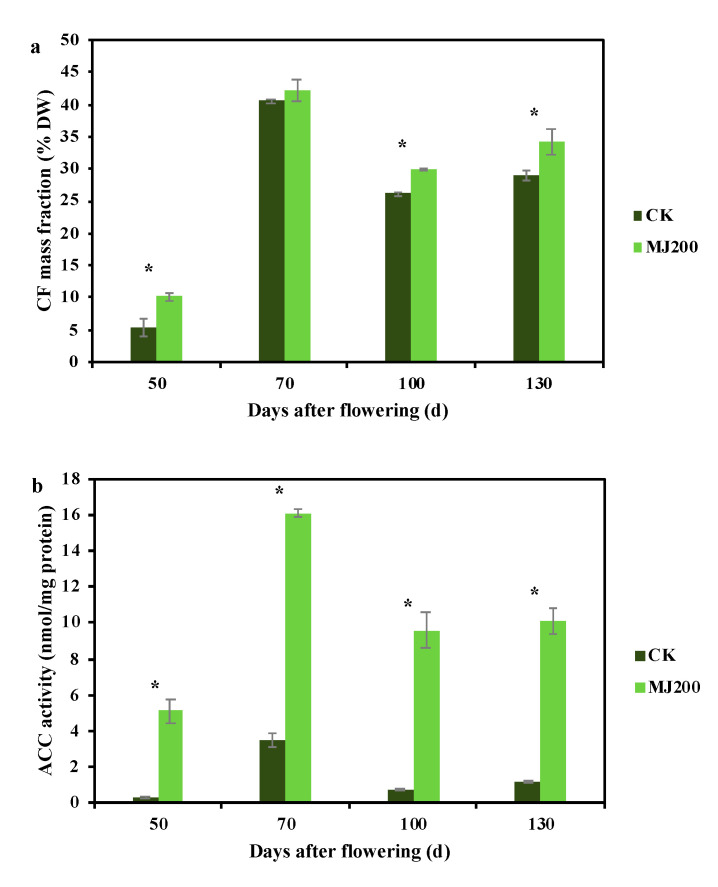

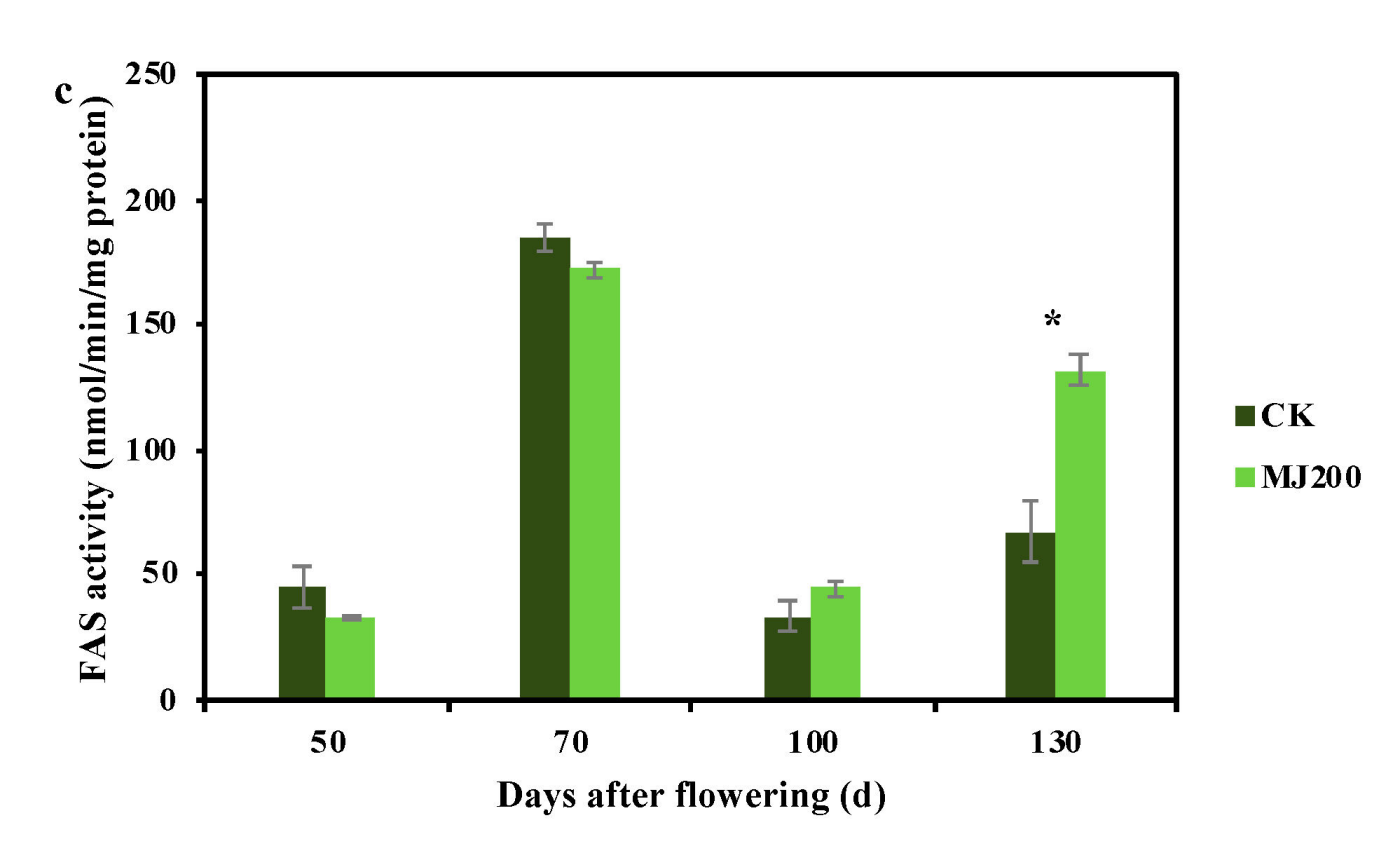
Effects of MJ200 on CF mass fraction and FA synthesis-related enzymes’ activity during seed development. (**a**) CF mass fraction in *S. tonkinensis* seeds at 50, 70, 100 and 130 DAF; (**b**,**c**) ACC and FAS activities in *S. tonkinensis* seeds at 50, 70, 100 and 130 DAF. Data points showed mean ± SD; * indicated significant difference (*p* < 0.05).

The application of 200 μmol/L of MJ (MJ200) obviously affected the CF mass fraction in *S. tonkinensis* seeds from the four sampling periods (Figure 1a). At 50 DAF, the CF mass fraction in the seeds from the MJ200-treated trees was 10.19%, which was significantly higher than that which was found in the seeds from untreated trees (CK seeds, 5.48%). Twenty days later, the CF mass fractions in the CK and MJ200 seeds were close, such that there was no significant difference between them, while the CF mass fraction was the highest at this point that it had been during the whole sampling period (40.51% in CK and 42.15% in MJ200). Thereafter, the CF mass fraction declined to 26.17% in the CK seeds and 30% in the MJ200 seeds at 100 DAF. At 130 DAF, the CF mass fraction had a slight increase and it reached up to 28.97% in the CK seeds and 34.29% in the MJ200 seeds. Overall, MJ200 promoted CF accumulation during the development of the seeds.

Concerning the activity of FA synthesis-related enzymes, the ACC activity displayed a similar trend to that of the CF mass fraction (Figure 1b). The ACC activity in the seeds from the MJ200-treated trees was significantly higher than that which was found in the CK seeds at all stages and the activity under the two treatments reached its maximum at 70 DAF, which was 3.48 nmol/mg protein and 16.12 nmol/mg protein. The dynamic of the fatty acid synthase (FAS) activity was obviously different from that of the ACC activity (Figure 1c). Although the FAS activity in the seeds from the CK- and MJ200-treated trees also touched the maximum at 70 DAF, the FAS activity in the CK seeds was higher than that which was found in the MJ200 seeds at this time, without significant difference. At 130 DAF, the FAS activity in the seeds from the MJ200-treated trees was 131.32 nmol/min/mg protein, exceeding 94.4% of that which was found in the CK seeds, and there was a significant difference between these results.

### 2.2. Dynamic Changing Trend of Fatty Acid Compositions under MJ200 Treatment

The dynamic in the concentration and composition of medium- and long-chain FA in the seeds from the CK and MJ200-treated trees are exhibited in Table 1. A total of 20 FAs were detected, including 10 saturated FAs (SFAs), mainly C16:0 and C18:0, as well as 10 UFAs, mainly C18:1, C18:2 and C18:3. The concentration of C16:0 in the seeds from the trees that were treated with MJ200 decreased continuously with the seeds’ development and the concentration of C16:0 in the CK seeds was slightly higher than that which was found in the MJ200 seeds. The concentration of C18:0 decreased first and then increased, reaching the highest level at 50 DAF. Among the UFAs, the concentrations of C16:1, C17:1, C20:1, C20:2, C20:3, C22:1 and C24:1 were extremely low and showed a trend of decreasing first and then increasing. The effects of MJ200 on the concentrations of these FAs were various. Among the main UFAs, the concentration of C18:2 was the highest in all periods and achieved its maximum at 70 DAF. The variation trend of the C18:1 concentration was similar to that of C18:2. The concentration of C18:1 was the lowest at 50 DAF and the highest at 70 DAF. The C18:3 concentration was lower than those of C18:1 and C18:2 and its concentration was 20.16% and 18.63% in the MJ200 and CK seeds at 50 DAF, respectively. In general, the concentrations of the UFAs were much higher than those of the SFAs and MJ200 had little effect on the concentrations and compositions of each fatty acid.

### 2.3. Analysis of Transcriptome Sequencing Data under MJ200 Treatment

#### 2.3.1. Expression Level of Differentially Expressed Genes

**Figure 2:**
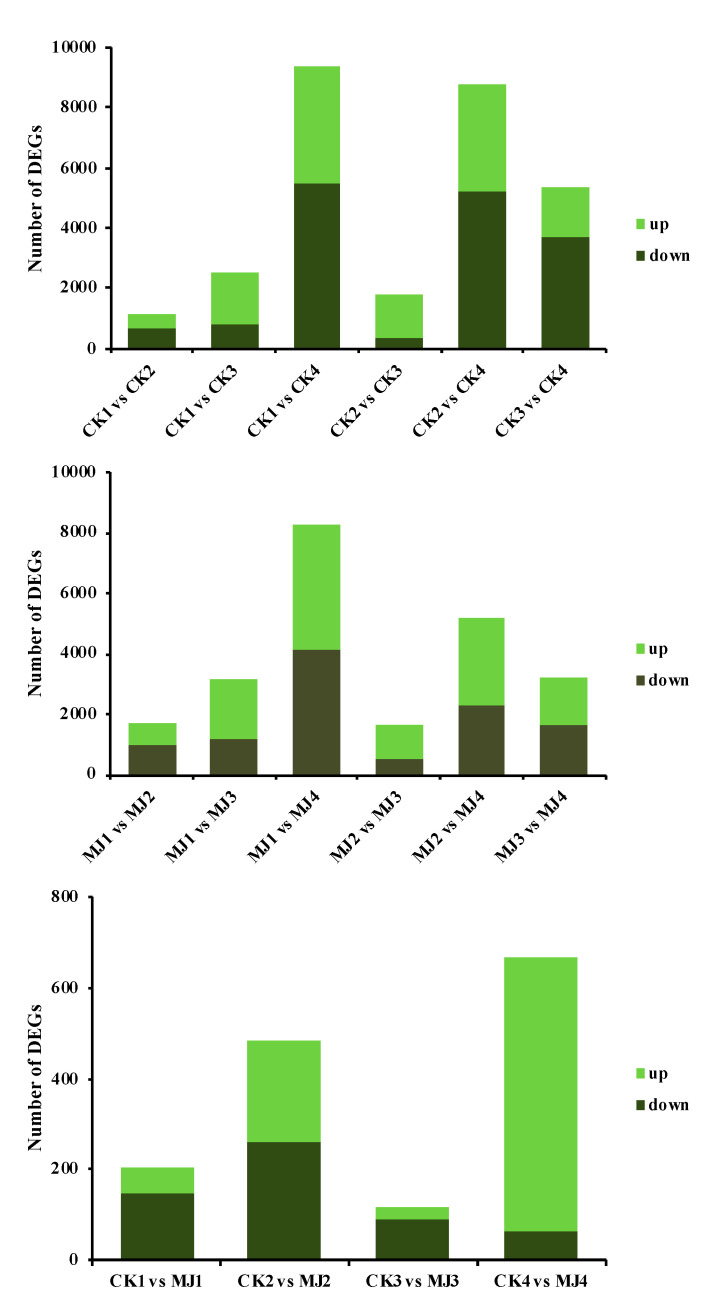
Comparison of the number of DEGs in CK and MJ200 seeds at different sampling times.

The changing trend of the number of differentially expressed genes (DEGs) within the seed development process is shown in Figure 2. Within this figure, the CK seeds that were used for the transcriptomic analyses at 50, 70, 100 and 130 DAF were denoted as CK1, CK2, CK3 and CK4, respectively and the MJ200 seeds that were used for transcriptomic analysis at 50, 70, 100 and 130 DAF were denoted as MJ1, MJ2, MJ3 and MJ4, respectively. Compared to 50 DAF, there were 1119, 2540 and 9389 DEGs at 70, 100 and 130 DAF, among which 5494 DEGs were upregulated and 3895 DEGs were downregulated between 50 and 130 DAF. A total of 8771 unigenes were expressed differentially between 70 and 130 DAF with 5196 upregulated unigenes and 3575 downregulated unigenes. During the last two sampling periods, both the number of upregulated unigenes and downregulated unigenes descended.

The number of DEGs in the MJ200 seeds was similar to that which was found in the CK seeds; that is, the DEGs number increased as the seed developed. Between 50 and 130 DAF, 8290 genes were counted, of which 4163 were upregulated and 4127 were downregulated. Whereas, when we compared the DEGs in the CK and MJ200 seeds, it was noted that the number of DEGs ascended first, then descended and then ascended with the development of seeds. Compared to the CK seeds at 130 DAF, the number of DEGs in the MJ200 seeds was the highest (668, with 65 upregulated genes and 603 downregulated genes).

#### 2.3.2. Enrichment Analysis of Differentially Expressed Genes

**Figure 3:**
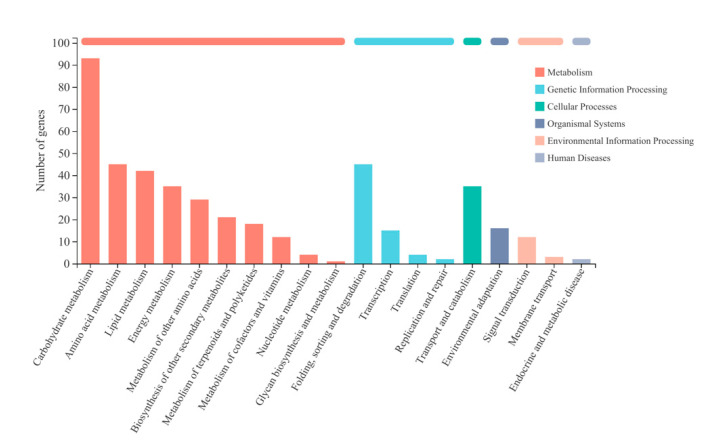
KEGG analysis of DEGs in *S. tonkinensis* seeds under MJ200 treatment.

The DEGs were subjected to Kyoto Encyclopedia of Genes and Genomes (KEGG) pathway enrichment analyses in order to screen for genes that are associated with FA biosynthesis in *S. tonkinensis* seeds. These DEGs were assigned into 6 KEGG categories, 19 sub-categories and 93 KEGG pathways (Figure 3 and Table S2). In the metabolism category, the carbohydrate metabolism, amino acid metabolism, lipid metabolism and energy metabolism were the major sub-categories. Next, we investigated the lipid metabolism classification, which was closely related to the purpose of this study. Among this classification, we found 11 KEGG pathways, including fatty acid degradation, fatty acid biosynthesis, cutin, suberine and wax biosynthesis, fatty acid elongation, glycerophospholipid metabolism, glycerolipid metabolism, steroid biosynthesis, arachidonic acid metabolism, linoleic acid metabolism, alpha-linolenic acid metabolism and the biosynthesis of unsaturated fatty acids.

#### 2.3.3. Identification and Expression Level of Genes Involved in Fatty Acid Biosynthesis

**Figure 4:**
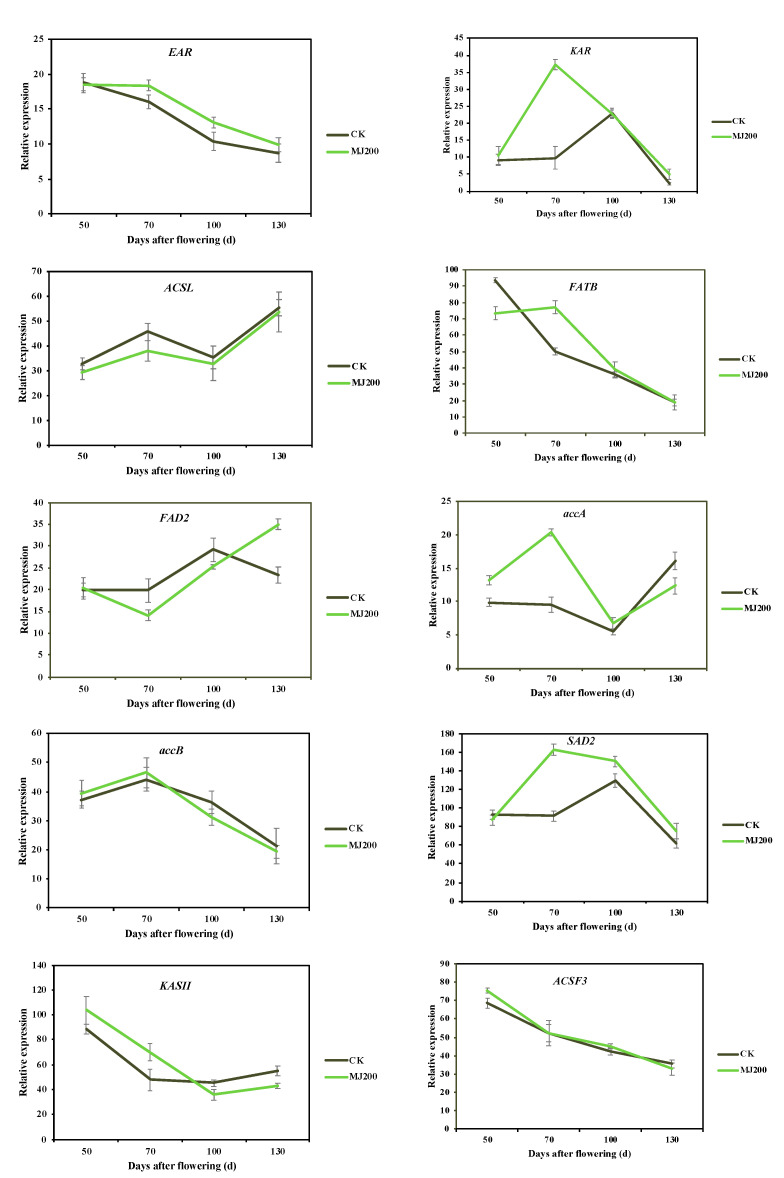
Effects of MJ200 on the expression of FA biosynthesis-related genes.

Based on the information that was obtained in the functional annotation, we selected 10 genes that are associated with FA biosynthesis and explored their expression patterns (Figure 4). The expression of long-chain acyl-CoA synthetases (*ACSL*) in the CK seeds was always higher than that which was found in the MJ200 seeds and it showed an upward–downward–upward trend. Meanwhile, the expression of acyl-ACP thioesterase B (*FATB*), which played a role in the saturated degree, type and quantity of the FAs, was higher at 50 and 70 DAF. FA desaturase 2 (*FAD2*) could catalyze the conversion of C18:1 to C18:2, which is crucial for the desaturation of UFAs. The trend of *FAD2* decreased first and then increased in both the CK- and MJ200-treated seeds. As for the expression level, it was generally higher in the CK seeds before 100 DAF. Stearoyl-ACP desaturase 2 (*SAD2*) was a key node in the FA biosynthesis pathway, which played a decisive role in the total FA concentration and the ratio of UFAs to SFAs. Enoyl-ACP reductase (*EAR*) and 3-ketoacyl-ACP reductase (*KAR*) were responsible for the FA carbon chain elongation in triacylglycerol biosynthesis. In addition, acc carboxyltransferase (*accA*) and acc biotin carboxyl (*accB*) were also rather important in FA biosynthesis. The dynamic of *EAR*, *KAR*, *accA*, *accB* and *SAD2* expression displayed an upward–downward trend during the seeds’ development and reached its maximum at 70 DAF, which was in agreement with the trend of the CF mass fraction, indicating that they were pivotal genes for FA biosynthesis in *S. tonkinensis* seeds. In general, MJ200 enhanced the *EAR*, *KAR* and *SAD2* expression in the seeds during the whole sampling period; nevertheless, *accA* and *accB* expression were promoted by MJ200 at 50 and 70 DAF. Unlike the genes that are mentioned above, the expression of 3-ketoacyl-ACP synthase II (*KASII*), which could increase the carbon chain and catalyze the formation of C16:0-ACP into C18:0-ACP, decreased first and then increased and MJ200 enhanced its expression at 50 and 70 DAF. Unlike *KASII*, the expression of acyl-CoA synthetase family member 3 (*ACSF3*) dropped continuously and MJ200 did not obviously affect its expression.

#### 2.3.4. Co-Expression Network Analysis

**Figure 5:**
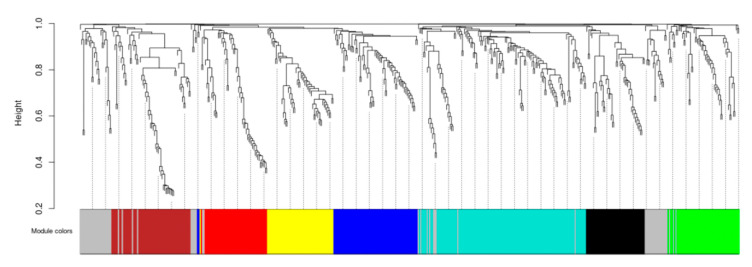
Hierarchical cluster tree displaying 8 co-expression modules. Each branch represents a gene and each color represents a module.

The accuracy of the weighted correlation network analysis (WGCNA) analysis was affected by genes with low expression levels or a small coefficient of variation. After pretreatment, genes from 24 samples were used for WGCNA analysis. By constructing a gene tree clustering map, eight modules were obtained (Figure 5).

**Figure 6:**
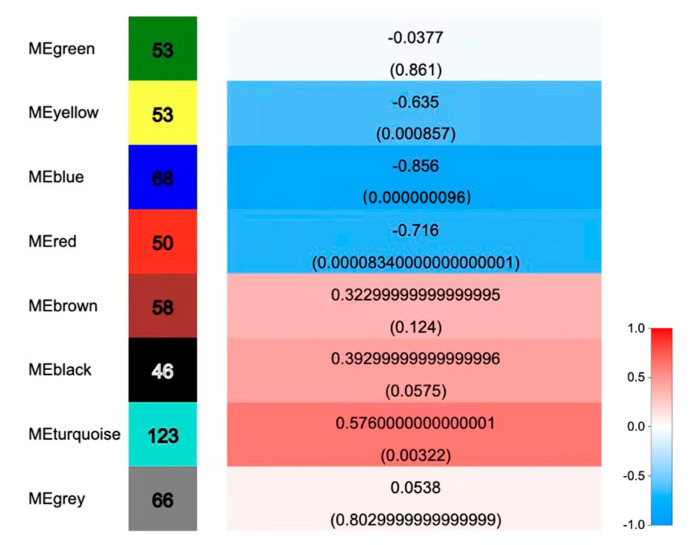
Correlations between WGCNA and crude fat mass fraction.

Correlation analysis was conducted between the genes in the modules and the phenotypes (the CF mass fraction in the seeds from the CK- and MJ200-treated trees) (Figure 6). The number of genes that were contained in each module ranged from 46 to 123, among which the MEturquoise module possessed the most genes (123), which was significantly positively correlated with the CF mass fraction (r = 0.576, *p* < 0.05). Both the MEbrown module (containing 58 genes) and the MEblack module (containing 46 genes) were positively correlated with the CF mass fraction, but these correlations were not significant (r = 0.322 and 0.392, respectively). Therefore, the genes in the MEturquoise module were closely related to the accumulation of CFs. The function of the genes in the MEturquoise module required further analysis and exploration. Several modules, such as MEgreen, MEyellow and MEred, were negatively correlated with the CF mass fraction.

**Figure 7:**
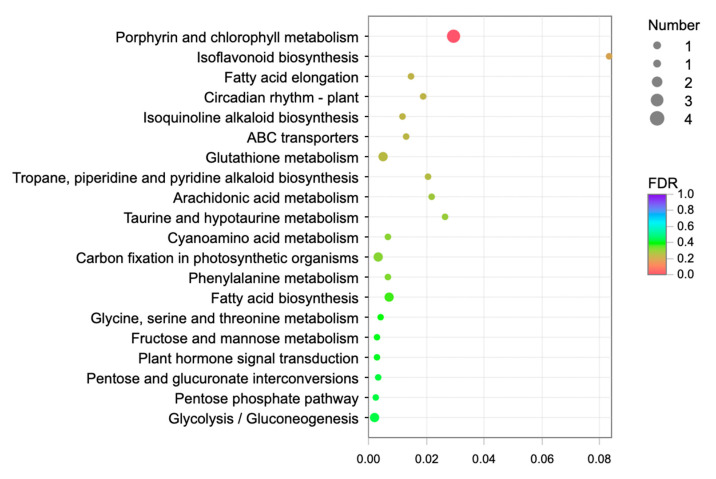
KEGG enrichment analysis of genes in module MEturquoise. The size of dots in the figure indicated the number of genes in this pathway, and the color of dots corresponded to different Qvalue ranges.

A KEGG enrichment analysis was performed for the genes in the MEturquoise module and the results of this are shown in Figure 7. Fatty acid biosynthesis, fatty acid elongation and arachidonic acid metabolism were the main biological pathways that were enriched in the lipid metabolism of the MEturquoise module. The pathway that enriched the most genes was the porphyrin and chlorophyll metabolism. Moreover, a large number of genes were also enriched in various metabolite synthesis pathways, suggesting that lipid accumulation may compete with the accumulation of other metabolites.

**Figure 8:**
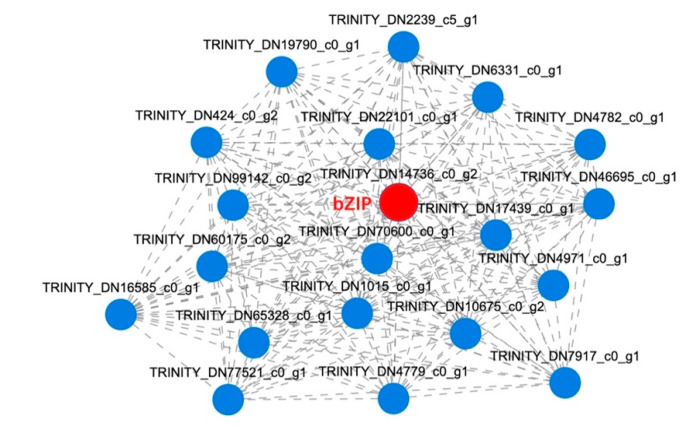
Co-expression network of hub genes in module MEturquoise.

“Core genes” refer to the genes with high connectivity in the co-expression module, which are the key genes in the co-expression network. The MEturquoise module was found to have played an important role in lipid metabolism. We studied the top 20 core genes in this module, especially those that are known to be related to FA biosynthesis, and then constructed a co-expression network through visualization (Figure 8). Located at the center of the co-expression network, *bZIP* had high connectivity, hence it was confirmed as a hub gene in the MEturquoise module. The expression level of *bZIP* in the MJ200 seeds was higher than that which was found in the CK seeds, suggesting that *bZIP* dominated in the FA biosynthesis process of *S. tonkinensis* seeds.

#### 2.3.5. Analysis of qRT-PCR Validation

According to the transcriptomic results, 10 genes that are related to FA biosynthesis were screened out for the quantitative real-time PCR (qRT-PCR) verification and their expression patterns are demonstrated in Figure S1. The expression of *KAR*, *EAR*, *accA*, *accB* and *SAD2* in the MJ200 seeds came up to their maximums at 70 DAF and MJ200 promoted their expression. The expression levels of *ACSF3* and *FATB* showed a general downward trend, while MJ200 promoted their expression at 50 or 70 DAF. Nonetheless, *ACSL*, *KASII* and *FAD2* were expressed variously. Generally, the qRT-PCR results were consistent with the transcriptomic sequencing results, indicating that the sequencing data were reliable.

## 3. Discussion

### 3.1. MJ200 Improved Crude Fat Mass Fraction and Fatty Acid Synthesis-Related Enzyme Activity

Biodiesel is a renewable, safe and green energy source, which is regarded as an alternative resource that may be able to alleviate the energy crisis [38,39]. After its extraction and processing, seed oil can be utilized as a raw material for the production of biodiesel. Previous studies have shown that *S. tonkinensis* seed contain abundant oil and excellent fuel properties [28]. As a result, artificial treatment helps to elevate seed oil, thereby realizing the industrialization of *S. tonkinensis*. In the present study, the CF mass fraction in the CK seeds increased first and then decreased, which may be caused by the carbon distribution between lipids and starches during the seeds’ development [27]. MJ200 enhanced the CF mass fraction in the CK seeds at each period (ranging from 4%–86%), indicating that the application of MJ200 favored CF accumulation, which agreed well with the results of existing research on *Camelina sativa* [40]. It was reported that the increase in FA synthesis-related enzymatic activity induced oil accumulation [41]. Our study demonstrated that ACC activity exhibited the same dynamic trend as the CF mass fraction and MJ200 significantly increased its activity, which collectively confirmed the above perspective. At 50 and 70 DAF, MJ200 decreased the FAS activity, but it increased the FAS activity at 100 and 130 DAF, implying that MJ200 had a greater effect on ACC activity when compared to the FAS activity. Hence, ACC could be the main FA synthesis-related enzyme in the oil accumulation of *S. tonkinensis*.

### 3.2. MJ200 Altered Fatty Acid Composition and Concentration

In addition to oil quantity, the proportion of FAs is critically important for feedstock quality [42,43]. Previous literature has stated that *S. tonkinensis* seeds are rich in UFAs that are required by the human body, but their concentration was not stable and their composition remained to be optimized [28]. In order to mitigate this deficiency, MJ200 was applied to *S. tonkinensis* in this study and a targeted metabolome was adopted in order to determine the FAs’ composition and concentration in the seeds. This process was aimed at revealing the accumulation pattern of the FAs, so as to provide a reference for extracting some beneficial FAs in production.

For edible oil, UFAs play a very important role in human health, so increasing their concentration is one of the key goals in edible oil production. If plant oil contains a high concentration of SFAs, its nutritional value is low. Inversely, if plant oil contains abundant UFAs, such as C18:1, and a certain proportion of C18:2 and C18:3 then the nutritional value of the oil is high. In the current study, a total of 20 FAs were detected in the seeds from the CK- and MJ200-treated trees, more than the 11 that were detected by [28]. The main UFAs that were detected in the CK seeds were C18:1, C18:2 and C18:3 and the concentrations of C18:1 and C18:2 increased in the MJ200 seeds at 70 DAF. Combined with the changing trend of the CF mass fraction, this was shown to be the optimal time to extract the seed oil and UFAs. The application of exogenous 2 μmol/L of MJ also significantly boosted the concentration of C18:2 in *Ribes nigrum* seeds [41]. Therefore, the MJ concentration that is required differs from species to species and this must be considered when designing an experiment. FAs with less than 20 carbon atoms were very similar to superior biodiesel components and could be used as raw materials in biodiesel production. Based on this, we found that 14 FAs in *S. tonkinensis* seeds satisfied the requirements for yielding biodiesel, which was higher than the number of FAs that are known to be present in *Ficus ulmifolia* and *Payenapara lleloneura* seeds [44,45]. Furthermore, it was reported that the mature seed oil of *S. tonkinensis* had good fuel characteristics and its density, kinematic viscosity, iodine value and cold filter plugging point met the standards of Germany, the European Union and the USA and it could be directly or simply transformed for biodiesel utilization [28]. To conclude, *S. tonkinensis* is a kind of excellent biodiesel species with nutritional value and oil value. During seed development, MJ200 treatment could apparently improve its seed oil value, thus promoting the multifunctional utilization of *S. tonkinensis*.

### 3.3. MJ200 Conduced the Expression Level of Fatty Acid Biosynthesis-Related Genes

Transcriptome analysis is a powerful tool for dissecting the full range of mRNA, tRNA, rRNA and other noncoding RNA molecules that are expressed by cells, organs or tissues in a specific environment or developmental stage [46]. With the help of transcriptomes, scientists have made significant advances in the study of plants’ stress resistance, secondary metabolism, photosynthesis and so on [47,48,49].

FA biosynthesis is a complex physiological and biochemical process that is fundamental to the production of membranes and lipids in plant plastids [50]. Clarifying the expression levels of various important genes in this process is helpful to promote the biosynthesis of FAs by molecular biological methods. *ACSL* mainly catalyzed the synthesis of acyl-CoA from SFAs with carbon atom numbers between 12 and 22 and induced the generation of long-chain FAs [51]. In *S. tonkinensis* seeds, *ACSL* were expressed highly at 70 and 130 DAF, while long-chain FAs’ (such as C18:0, C20:0, C20:1 and C22:0) concentrations were low at these two periods, suggesting that there might be negative regulatory genes affecting the biosynthesis of long-chain FAs in the seeds at 70 and 130 DAF. Furthermore, their expression levels were notably higher than that of *ACSL*. The overexpression of *ACSL9* in *Elaeis guineensis* embryoids increased the C20:1, C20:2 and C24:0 concentrations [52]. Besides this, *ACSL* prompted *A. thaliana* into early flowering and enhanced its abiotic stress resistance [53]. Consequently, it was deemed worth exploring whether *ACSL* has broader biological functions in *S. tonkinensis* seeds. *FATB* is expressed variously in all of the higher plants and has been verified to participate in producing SFAs in *A. thaliana* flowers and seeds [54,55]. Its expression was relatively high at 50 and 70 DAF and it declined substantially at 100 and 130 DAF, basically coinciding with the concentration of C16:0. MJ200 generally rose *FATB* expression and could be considered as an effective way to improve SFA concentration in *S. tonkinensis* seeds. KARs were found to be indispensable components of the plant’s FAS complex system, which could reduce the β-ketoacyl-ACP product resulting from condensations during the creation of acyl carbon chains. It was well documented that *KAR* genes functioned prominently in *de novo* FAs’ biosynthesis in *Helianthus annuus* seeds [56]. Similarly, *KAR* also profoundly affected the biosynthesis of FAs in the *S. tonkinensis* seeds. Specifically, the expression of *KAR* showed a drastic uptrend at 70 DAF while, simultaneously, the activity of the FAS remained at a plateau level, illustrating that *KAR* might raise the FAs’ concentrations by enhancing the activity of the enzymes that are related to FA biosynthesis. MJ200 increased the level of *KAR* expression, thus it was concluded that MJ200 had a great effect on the accumulation of FAs in *S. tonkinensis* seeds, both at the physiological and molecular level. As a vital enzyme in the synthesis and metabolism of UFAs, *SAD* not only catalyzed the dehydrogenation of C18:0 to C18:1, but it also played a decisive role in the total FA concentration and the ratio of UFAs to SFAs [57,58]. In each sampling period, the concentration of C18:0 was much lower than that of C18:1. The possible reason was that *SAD* catalyzed the formation of C18:1 with C18:0 as the substrate. Moreover, MJ200 increased the concentration of C18:1 at 70 and 130 DAF. It is well known that C18:1 is an important UFA that is good for human health. Future studies could attempt to overexpress the *StSAD* gene and then examine the change in the C18:1 concentration, which could improve the value of *S. tonkinensis* seed oil.

WGCNA analysis can cluster together genes with similar expression patterns and then construct a gene co-expression network in order to obtain gene modules with common expression patterns. Next, the relationship between the key gene modules and the concerned phenotype may be established in order to explore the correlation between them and the essential genes in the co-expression network can be deeply mined, as presented in [59]. In order to study the expression patterns of the genes that are related to FA biosynthesis in *Artemisia sphaerocephala* seeds, WGCNA was used to construct a gene co-expression network and the gene expression patterns were divided into 7 modules, among which the MEblack module possessed a high correlation with *A. sphaerocephala* seeds at 20 DAF (r > 0.4, *p* < 0.05). Further analysis showed that Fusca 3 (*FUS3*) and a basic helix–loop–helix (*bHLH*) in MEblack were co-expressed with biotin carboxyl carrier protein (*BCCP*), 3-hydroxyacyl-ACP dehydratase (*HAD*), *EAR* and *FATB* as well as other genes that regulate FA biosynthesis. As such, *FUS3* and *bHLH* were found to be the core genes in the FA accumulation of *A. sphaerocephala* seeds [5]. In our experiment, eight modules were obtained through WGCNA, among which the *bZIP* gene in the MEturquoise module was identified as one of the core genes that is involved in the regulation of FA biosynthesis. Extensive work elucidated that *bZIP* was capable of boosting the oil content and altering the FA compositions in *A. thaliana* [15,60]. As a consequence, a study on the expression of *bZIP* would assist in deepening the understanding of the FA biosynthesis mechanism that is present in *S. tonkinensis* seeds. Glycerol-3-phosphate acyltransferase (*GPAT*) and NADPH oxidase (*NOX*) were closely associated with triacylglycerol synthesis and oleic acid metabolism, respectively [61]. We hypothesized that the FA biosynthesis process of *S. tonkinensis* must be regulated by numerous genes that are responsible for signal transduction, energy metabolism and so on [62]. These different expression levels of genes have been found to have interacted in order to form a complex biomolecular regulatory network, which further manipulated the synthesis, accumulation and metabolism of FA metabolites [63,64].

Hence, exogenous MJ200 treatment is an efficient, environmentally friendly and simple method to promote FA accumulation by enhancing the expression of genes that are related to FA biosynthesis. Our experimental data provide a basis for determining the expression levels of FA biosynthesis-related genes at different developmental stages and for further studying the molecular mechanism of FA biosynthesis in and the component improvement of *S. tonkinensis* seeds.

## 4. Materials and Methods

### 4.1. Plant Materials and Experimental Design

The *S. tonkinensis* (which originated from Jishui, Jiangxi Province) were planted in the Styracaceae Germplasm Repository, Luhe District, Nanjing, China (32°54′ N, 118°50′ E). In our previous experiments, we had manipulated four concentrations of MJ (10, 50, 200 and 500 μmol/L) so as to spray the whole trees in order to study their effect on the FA accumulation in the trees’ seeds. Based on the results of this experiment, we found that the FA concentration in seeds from MJ-treated trees peaked at 70 DAF and that 200 μmol/L MJ (marked as MJ200) boosted FA accumulation in each period. Therefore, we selected the samples that were treated with MJ200 for transcriptome and targeted metabolome examination. On 1 July 2020 (45 DAF), approximately 1800 mL of MJ (diluted with distilled water into 200 μmol/L) was sprayed on six sampled trees (i.e., sprayed on the whole tree) and another six trees were treated with an equal amount of distilled water (this group is referred to in this study as CK). This exogenous treatment was conducted every ten days thereafter until 135 DAF and the process was always performed between 6:00 and 8:00 a.m.

### 4.2. Sample Collection

On the basis of our previous research on the seed development of *S. tonkinensis* [27,28], we selected samples from four representative time points:

- 50 DAF, the previous stage before the seeds’ dry matter rapidly increases
- 70 DAF, during the aforementioned steep rise in nutrient concentration
- 100 DAF, the stage that is marked by decreasing oil concentration and increasing starch concentration
- 130 DAF, the final maturation stage

Ten seeds were randomly collected from each individual tree at each sampling time and the seeds that were from the same treatment group were mixed. These samples were placed in an insulated box with dry ice for transport to the laboratory. Forty-five seeds were used for the CF mass fraction determination and fifteen seeds were used for the FAS and ACC assays. Besides these, the other five seeds from each tree were sampled and every two trees were regarded as one biological replicate, so three biological replicates in each treatment with ten seeds in each replicate were used for the transcriptome analysis. With respect to the metabolome analysis, each tree was considered to be a biological replicate and ten seeds were sampled from each replicate. These samples for transcriptome and metabolome analysis were immediately frozen in liquid nitrogen and stored at −80 °C until they were used.

### 4.3. Crude Fat Extraction

Forty-five seeds, divided into three replicates, were dried at 65 °C for 72 h. Then, these seeds were crushed and transferred into filter paper, wherein they were dried and weighed (M_0_). The seeds and filter paper were weighed again (M_1_) after drying for 30 min. Petroleum ether (30–60 °C) was used as the extraction solution and a Soxhlet apparatus was adopted in order to extract the CF. The extraction process lasted for 72 h and the water temperature was controlled at 65 °C. After the extraction, the filter paper that was covering the seeds was dried at 65 °C for 30 min (M_2_). The CF mass fraction was calculated according to the formula below:

$$\mathrm{CF}\;\mathrm{mass}\;\mathrm{fraction}\;\left(\%\right)=\frac{M_{1}{\;-\;M}_{2}}{M_{1}{\;-\;M}_{0}}\;\times \;100\%$$

### 4.4. Targeted Metabolome Analysis

A total of 0.2 g of solid seeds was accurately weighed and the FA compositions of these were extracted using 0.5 mL of methanol and 0.5 mL of chloroform with a steel ball. The mixture was homogenized at 50 Hz for 3 min. After being set for 15 min, the mixture was centrifuged at 13,000 rpm for 10 min. Then, 1 mL of dichloromethane was added into the supernatant and then the above steps were repeated. After this repetition, 1 mL of sodium hydroxide–methanol solution was added to the mixed solution, which was then shaken for 30 s and places in a 60 °C water bath for 30 min. Then, 1 mL of n-hexane and 1 mL of sodium hydroxide were added after the water bath was cooled. The mixture was centrifuged at 13,000 rpm for 10 min. The upper solution was then transferred to a 1.5 mL centrifuge tube, 2 mL of n-hexane was added and the solution was shaken for 30 s. The mixture was then centrifuged at 13,000 rpm for 5 min at 4 °C. The supernatant was transferred into GC vials. The GC–MS analysis was performed on an Agilent 8890B-5977B GC–MS system (Agilent Technologies Inc., Santa Clara, CA, UAS) coupled with an Agilent DB-FastFAME column (20 m, 0.18 mm, 0.2 μm, Agilent J&W Scientific, Folsom, CA, USA). Before sample examination, standard substances—(GLC Reference Standard (Fatty acid methyl ester) and GLC-674 (52 components, C4–C24)—that were obtained from Anpel Laboratory Technologies Inc., Songjiang, Shanghai, China) with different concentrations for each FA methyl ester were used in order to make a standard curve for the quantification of the FA concentration in the sample.

The GC conditions were as follows: helium (purity > 99.999%) was used as carrier gas at a flow of 1 mL/min. The injection volume of the sample was 1 μL and introduced in splitting mode (50:1) with an injector temperature of 250 °C. The GC column temperature was programmed to hold at 80 °C for 0.5 min and rose to 180 °C at a rate of 70 °C per minute, then increased to 220 °C at a rate of 4 °C per minute and finally it was held at a temperature of 240 °C for 2 min. The MS conditions were as follows: an electron ionization system was used with 70 eV of ionization energy. The ion source’s temperature was 230 °C, the quadrupole’s temperature was 150 °C and the transmission line’s temperature was 240 °C. Data acquisition was conducted on selective ion scan mode. The compounds were identified and quantified by the software of Masshunter (v10.0.707.0, Agilent Technologies Inc., Santa Clara, CA, USA) with manual inspection. A linear regression standard curve was made with the mass spectrum peak area of the analyte as the ordinate and the concentration of the analyte as the abscissa. The mass spectrum peak area of the analyte was substituted into the linear equation in order to calculate the sample’s concentration.

### 4.5. Enzyme Activity Assay

For this assay, 0.5 g of seeds was weighed in order to be able to measure the FAS activity with an assay kit (Suzhou Comin Biotechnology Co., Ltd., Suzhou, Jiangsu, China). As described by [65], the samples were homogenized in a buffer containing 1 mmol/L EDTA-Na_2_, 2 mmol/L AsA and 20 mmol/L Tris-HCl (pH 7.5). The reactions were performed in a total volume of 1000 μL (100 μL of supernatant, 20 μL of acetyl-CoA, 20 μL of malonyl-CoA, 820 μL of 30 °C PBS (pH 7.0) and 40 μL of NADPH). Decreases in the NADPH absorbance at 340 nm were detected with a spectrophotometer and the activity of the FAS was calculated by the use of the method that is described in [66].

An enzyme-linked immunosorbent assay (ELISA) kit (Suzhou Comin Biotechnology Co., Ltd.) was used for analyzing the activity of the ACC. For this analysis, 0.5 g of seeds was weighed and ground with extraction solution and then centrifuged at 20,000 rpm for 10 min at 4 °C. The supernatant was used for the subsequent determination. The microtiter plates had been pre-coated with an antibody that is specific to plant ACC. After the antibody–antigen interactions, the signals were amplified by the biotin–avidin system and the antigen targets in the samples were qualified through the HRP (horseradish peroxidase)-TMB (3,3′,5,5′-tetramethylbenzidine) colorimetric detection system. The color change was measured spectrophotometrically at a wavelength of 450 nm. The ACC activity in the samples was then determined by comparing the OD values to the standard curve.

### 4.6. Transcriptome Analysis

Total RNA was extracted from the kernels’ tissues using Plant RNA Purification Reagent (Invitrogen, Carlsbard, CA, USA) according to the manufacturer’s instructions, then the integrity and purity of the total RNA quality was determined by the use of a 2100 Bioanalyser (Agilent Technologies, Inc., Santa Clara, CA, USA) and quantified using the ND-2000 (NanoDrop Thermo Scientific, Wilmington, DE, USA). An Illumina NovaSeq 6000 instrument (Illumina, San Diego, CA, USA) was adopted for sequencing. The clean data that were obtained were used to do de novo assembly with Trinity. Moreover, the KEGG pathway analysis was carried out by Kobas [67]. Genes with expression levels that were less than 1 and coefficients of variation that were less than 0.1 were removed for WGCNA. Ten genes that were related to FA biosynthesis were selected for validation using qRT-PCR with a StepOne Real-Time PCR System (Applied Biosystems, Foster City, CA, USA) and SYBR Green Premix Pro Taq HS qPCR Kits (AG11701, Accurate Biotechnology Co., Ltd., Changsha, Hunan, China). The relative gene expression was calculated by the 2^−ΔΔCt^ method with 18S ribosomal RNA as an internal control. All of the primers that were used in this study are listed in Table S1.

### 4.7. Statistical Analysis

The values were expressed as mean ± SD for the three replicates. Excel (Office 2019 Pro Plus, Microsoft Corporation, Redmond, WA, USA) was used to process the data. A one-way analysis of variance (ANOVA) was performed using SPSS 26.0 (IBM, Armonk, NY, USA) and this was then followed by Duncan’s multiple range test. In considering these results, *p*-values of less than 0.05 were considered to indicate significance within the groups.

## 5. Conclusions

In this study, MJ200 promoted the accumulation of CF and the concentration of primary FAs in *S. tonkinensis* seeds. The increase in the activity of the enzymes that are related to FA synthesis supplied solid physiological evidence that MJ200 could improve FA accumulation. *EAR*, *KAR*, *SAD2*, *accA*, *accB* and *bZIP, as* screened by WGCNA, were beneficial to FA biosynthesis. Our results clearly interpreted the physiological and molecular mechanism of FA biosynthesis in *S. tonkinensis* seeds under the intervention of exogenous MJ200. The experimental data could provide reference for the scientific and accurate exploitation of the oil value of *S. tonkinensis*.

## Figures and Tables

**Table 1:** Effects of MJ200 on FA compositions during seed development.

FA Composition (%)								
	50 DAF		70 DAF		100 DAF		130 DAF	
	CK	MJ200	CK	MJ200	CK	MJ200	CK	MJ200
C14:0	0.17 ± 0.06	0.14 ± 0.05	0.08 ± 0.04	0.07 ± 0.02	0.08 ± 0.05	0.09 ± 0.04	0.08 ± 0.06	0.08 ± 0.03
C15:0	0.10 ± 0.02	0.05 ± 0.01	0.04 ± 0.02	0.04 ± 0.01	0.02 ± 0.01	0.02 ± 0.02	0.02 ± 0.01	0.02 ± 0.01
C16:0	25.53 ± 2.02	22.55 ± 3.11	17.29 ± 3.15	16.18 ± 1.33	11.73 ± 0.94	11.93 ± 2.08	10.41 ± 2.26	10.26 ± 1.77
C16:1	0.24 ± 0.02	0.24 ± 0.02	0.14 ± 0.04	0.13 ± 0.02	0.18 ± 0.01	0.15 ± 0.06	0.13 ± 0.08	0.14 ± 0.05
C17:0	0.64 ± 0.03	0.67 ± 0.04	0.79 ± 0.32	0.73 ± 0.07	0.43 ± 0.16	0.45 ± 0.12	0.37 ± 0.11	0.35 ± 0.03
C17:1	0.41 ± 0.03	0.33 ± 0.01	0.37 ± 0.18	0.40 ± 0.03	0.31 ± 0.10	0.28 ± 0.02	0.24 ± 0.06	0.22 ± 0.04
C18:0	5.95 ± 0.78	6.39 ± 0.48	3.17 ± 0.74	2.80 ± 0.21	2.58 ± 1.27	3.28 ± 1.93	3.79 ± 1.57	3.71 ± 0.55
C18:1	13.80 ± 1.30	13.39 ± 1.12	29.36 ± 2.00	29.93 ± 3.29	23.33 ± 2.65	22.28 ± 2.44	11.65 ± 2.06	11.71 ± 1.33
C18:2	24.86 ± 1.70	24.74 ± 1.60	53.86 ± 5.04	55.09 ± 5.21	53.43 ± 6.00	52.14 ± 5.60	44.25 ± 7.00	44.43 ± 4.98
C18:3	18.63 ± 1.85	20.16 ± 1.31	8.52 ± 1.50	7.81 ± 0.61	6.89 ± 1.17	7.81 ± 1.88	9.99 ± 2.62	9.65 ± 1.57
C20:0	2.30 ± 0.22	2.67 ± 0.20	0.85 ± 0.16	0.90 ± 0.05	0.24 ± 0.14	0.41 ± 0.15	0.38 ± 0.13	0.38 ± 0.11
C20:1	1.45 ± 0.25	2.14 ± 0.28	0.61 ± 0.24	0.63 ± 0.03	0.24 ± 0.08	0.30 ± 0.05	0.37 ± 0.18	0.33 ± 0.10
C20:2	0.41 ± 0.01	0.71 ± 0.03	0.22 ± 0.01	0.50 ± 0.01	0.06 ± 0.03	0.09 ± 0.07	0.05 ± 0.01	0.05 ± 0.02
C20:3	0.37 ± 0.01	0.48 ± 0.01	0.20 ± 0.01	0.30 ± 0.01	0.03 ± 0.01	0.04 ± 0.01	0.02 ± 0.02	0.02 ± 0.01
C21:0	0.61 ± 0.02	0.67 ± 0.03	0.30 ± 0.04	0.33 ± 0.01	0.04 ± 0.02	0.06 ± 0.03	0.04 ± 0.01	0.03 ± 0.01
C22:0	1.72 ± 0.22	1.57 ± 0.08	0.65 ± 0.11	0.70 ± 0.02	0.16 ± 0.01	0.25 ± 0.13	0.20 ± 0.03	0.18 ± 0.03
C22:1	0.17 ± 0.06	0.10 ± 0.04	0.06 ± 0.04	0.07 ± 0.04	0.05 ± 0.03	0.04 ± 0.01	0.02 ± 0.01	0.02 ± 0.01
C23:0	0.54 ± 0.02	0.67 ± 0.02	0.28 ± 0.03	0.40 ± 0.01	0.06 ± 0.03	0.08 ± 0.06	0.06 ± 0.01	0.05 ± 0.01
C24:0	1.62 ± 0.16	1.67 ± 0.07	0.71 ± 0.11	0.87 ± 0.04	0.14 ± 0.10	0.25 ± 0.15	0.17 ± 0.08	0.14 ± 0.10
C24:1	0.47 ± 0.01	0.67 ± 0.01	0.28 ± 0.02	0.43 ± 0.01	0.06 ± 0.04	0.06 ± 0.01	0.04 ± 0.02	0.02 ± 0.02

Note: C14:0—tetradecanoic acid, C15:0—pentadecanoic acid, C16:0—palmitic acid, C16:1—palmitoleic acid, C17:0—heptadecanoic acid, C17:1—ginkgolic acid, C18:0—stearic acid, C18:1—oleic acid, C18:2—linoleic acid, C18:3—linolenic acid, C20:0—eicosanoic acid, C20:1—cis-11-eicosenic acid, C20:2—cis-11,14-eicosadienoic acid, C20:3—cis-11,14,17-eicosatrienoic acid, C21:0—n-heneicosanoic acid, C22:0—docosanoic acid, C22:1—erucic acid, C23:0—tricosanoic acid, C24:0—lignoceric acid and C24:1—nervonic acid.

## Data Availability

Raw sequence data were deposited in the NCBI Short Read Archive database under accession number PRJNA834066 and PRJNA791699.

## Supplementary Materials

The following supporting information can be downloaded at: https://www.mdpi.com/article/10.3390/ijms23116190/s1.
