# Supplementary figures and images for: Transcriptomic and Metabolomic Analysis Unravels the Molecular Regulatory Mechanism of Fatty Acid Biosynthesis in Styrax tonkinensis Seeds under Methyl Jasmonate Treatment
Source: Int J Mol Sci. 2022 May 31;23(11):6190. doi: 10.3390/ijms23116190 (PMC9181076; doi:10.3390/ijms23116190)

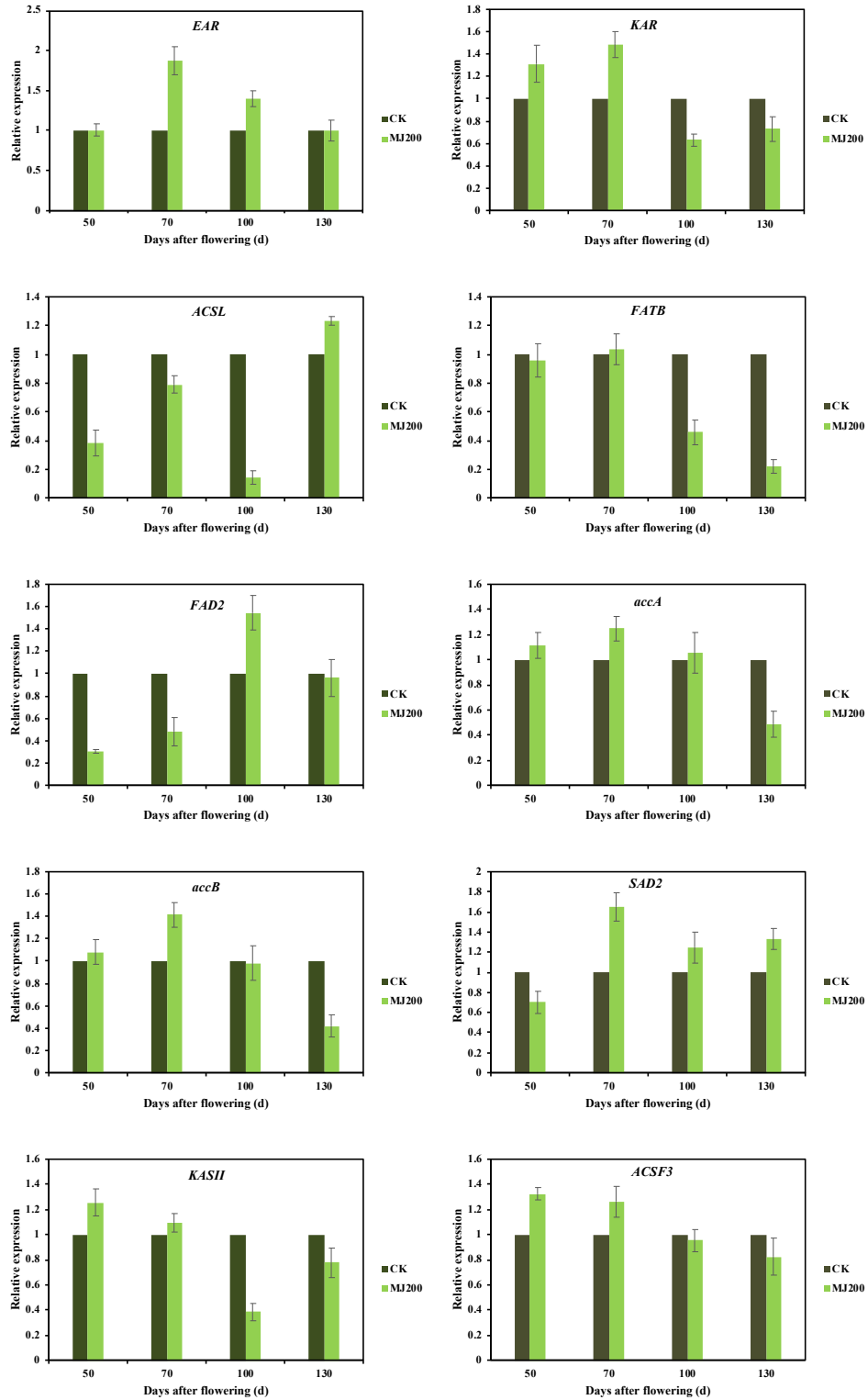

Figure S1. Validation of genes expression patterns uncovered by RNA-Seq

Supplement: Supplementary file 1 [file ijms-23-06190-s001.zip › Figure S1.pdf]
